# Efficacy of live attenuated and inactivated influenza vaccines among children in rural India: A 2-year, randomized, triple-blind, placebo-controlled trial

**DOI:** 10.1371/journal.pmed.1003609

**Published:** 2021-04-29

**Authors:** Anand Krishnan, Lalit Dar, Siddhartha Saha, Venkatesh Vinayak Narayan, Rakesh Kumar, Ramesh Kumar, Ritvik Amarchand, Shivram Dhakad, Reshmi Chokker, Avinash Choudekar, Giridara Gopal, Aashish Choudhary, Varsha Potdar, Mandeep Chadha, Kathryn E. Lafond, Stephen Lindstrom, Marc-Alain Widdowson, Seema Jain

**Affiliations:** 1 Centre for Community Medicine, All India Institute of Medical Sciences, Delhi, India; 2 Microbiology Department, All India Institute of Medical Sciences, Delhi, India; 3 Influenza Program, Centers for Disease Control and Prevention, New Delhi, India; 4 National Institute of Virology, Pune, India; 5 Influenza Division, Centers for Disease Control and Prevention, Atlanta, Georgia, United States of America; The University of Sheffield, UNITED KINGDOM

## Abstract

**Background:**

Influenza is a cause of febrile acute respiratory infection (FARI) in India; however, few influenza vaccine trials have been conducted in India. We assessed absolute and relative efficacy of live attenuated influenza vaccine (LAIV) and inactivated influenza vaccine (IIV) among children aged 2 to 10 years in rural India through a randomized, triple-blind, placebo-controlled trial conducted over 2 years.

**Methods and findings:**

In June 2015, children were randomly allocated to LAIV, IIV, intranasal placebo, or inactivated polio vaccine (IPV) in a 2:2:1:1 ratio. In June 2016, vaccination was repeated per original allocation. Overall, 3,041 children received LAIV (*n* = 1,015), IIV (*n* = 1,010), nasal placebo (*n* = 507), or IPV (*n* = 509). Mean age of children was 6.5 years with 20% aged 9 to 10 years.

Through weekly home visits, nasal and throat swabs were collected from children with FARI and tested for influenza virus by polymerase chain reaction. The primary outcome was laboratory-confirmed influenza-associated FARI; vaccine efficacy (VE) was calculated using modified intention-to-treat (mITT) analysis by Cox proportional hazards model (PH) for each year.

In Year 1, VE was 40.0% (95% confidence interval (CI) 25.2 to 51.9) for LAIV and 59.0% (95% CI 47.8 to 67.9) for IIV compared with controls; relative efficacy of LAIV compared with IIV was −46.2% (95% CI −88.9 to −13.1). In Year 2, VE was 51.9% (95% CI 42.0 to 60.1) for LAIV and 49.9% (95% CI 39.2 to 58.7) for IIV; relative efficacy of LAIV compared with IIV was 4.2% (95% CI −19.9 to 23.5). No serious adverse vaccine-attributable events were reported. Study limitations include differing dosage requirements for children between nasal and injectable vaccines (single dose of LAIV versus 2 doses of IIV) in Year 1 and the fact that immunogenicity studies were not conducted.

**Conclusions:**

In this study, we found that LAIV and IIV vaccines were safe and moderately efficacious against influenza virus infection among Indian children.

**Trial registration:**

Clinical Trials Registry of India CTRI/2015/06/005902.

## Abstract 2

Anand Krishnan and co-workers study the efficacy and safety of influenza vaccines for children in India.

## Author summary

**Why was this study done?:**

- In recent years, 2 community-based trials of the single-dose Russian-backbone live attenuated influenza vaccine (LAIV) in low- and middle-income countries (LMIC) have been published, with contrasting results.
- In Senegal, LAIV vaccine efficacy (VE) was demonstrated to be 0.0% (95% confidence interval (CI) −26.4 to 20.9), and in Bangladesh, LAIV VE was demonstrated to be 41.0% (95% CI 28.0 to 51.6) against all influenza virus strains. In addition, observational studies for VE of LAIV such as in the United States and Europe have also shown mixed results.
- While the World Health Organization (WHO) continues to recommend LAIV use in children, conflicting data on LAIV have led countries to review and reconsider national preferential recommendations for LAIV use in children.
- Given these gaps in evidence and the potential for indigenously produced LAIV to be used for prevention of influenza virus infection in Indian children, we evaluated the absolute efficacy of LAIV and inactivated influenza vaccine (IIV) and relative efficacy of LAIV versus IIV among children aged 2 to 10 years in rural India.

**What did the researchers do and find?:**

- We conducted a 2-year, triple (participant–observer–analyst) blind, community-based vaccine trial. We randomly assigned 3,041 children aged 2 to 10 years to receive age appropriate doses of LAIV, IIV, or a control vaccine.
- In Year 1, VE was 40.0% (95% CI 25.2 to 51.9) for LAIV and 59.0% (95% CI 47.8 to 67.9) for IIV compared with controls; relative efficacy of LAIV compared with IIV was −46.2% (95% CI −88.9 to −13.1). In Year 2, VE was 51.9% (95% CI 42.0 to 60.1) for LAIV and 49.9% (95% CI 39.2 to 58.7) for IIV; relative efficacy of LAIV compared with IIV was 4.2% (95% CI −19.9 to 23.5). We found that LAIV and IIV were safe and moderately efficacious against laboratory-confirmed influenza virus infection. In Year 1, LAIV, as a single dose in a vaccine-naïve population, was significantly less efficacious than IIV, whereas in Year 2, VE for LAIV and IIV was similar.
- This was the first randomized controlled trial to our knowledge in a developing country setting that compared LAIV efficacy with IIV and provided VE by age group and influenza type/subtype across seasons spanning 2 consecutive years. Importantly, during both years, LAIV and IIV were protective against influenza A(H3N2) viruses. However, LAIV had limited efficacy against influenza A(H1N1)pdm09 for both years.

**What do these findings mean?:**

- Influenza vaccination is an important tool for preparedness and response against seasonal epidemics and pandemics.
- However, decisions on specific vaccine introductions into national immunization platforms depend on multiple factors including effectiveness, presence of existing immunization platforms and cold chain infrastructure, cost, and local production of the vaccines.
- Availability of indigenous, safe, and moderately efficacious influenza vaccines, including LAIV, should initiate discussion on the optimal use of vaccines for the national strategy for influenza prevention and control in India.

## Introduction

Influenza causes significant pediatric mortality and morbidity globally, including in India [1–3]. Vaccines, including inactivated influenza vaccine (IIV) and live attenuated influenza vaccine (LAIV) formulations, are key to influenza prevention and control and have demonstrated moderate effectiveness when matched to circulating strains [4]. A recent 3-year trial of trivalent IIV among children aged 6 months to 10 years in India reported varying efficacy ranging from 26% (95% confidence interval (CI) 7 to 41) in the first year (2009 to 2010) to 74% (95% CI 58 to 84) in the third year (2011 to 2012) [5]. However, LAIV has several potential advantages in resource-poor settings, also applicable during pandemics, such as easier to administer intranasally versus injection; requirement for less antigen during manufacturing as compared with IIV; and potential protection with only 1 Russian-backbone LAIV dose [6]. Therefore, in 2006, the World Health Organization (WHO) included LAIV in its Global Action Plan for Influenza Vaccines [7]. Furthermore, a review of 4 trials conducted before 2009 among children aged 6 months to 18 years demonstrated LAIV to be more efficacious than IIV [4]. Thus, in 2009, WHO granted sublicenses to vaccine manufacturers in developing countries, including the Serum Institute of India (SII, Pune, India), for the development, manufacture, use, and sale of the egg-based LAIV using Russian master donor viruses [8].

Two recent community-based placebo-controlled trials of the single-dose Russian-backbone LAIV conducted in Senegal and Bangladesh using the same lot of vaccine reported contrasting results. In Senegal, LAIV vaccine efficacy (VE) was 0.0% (95% CI −26.4 to 20.9) [9], and in Bangladesh, LAIV VE was 41.0% (95% CI 28.0 to 51.6) against all influenza virus strains [6]. The reasons for the different findings are unclear and likely multifactorial. In parallel, observational studies in the United States indicated that LAIV did not perform as well as the IIV [10], particularly for influenza A(H1N1)pdm09 virus among children aged 2 to 17 years [11].

Based on these US findings, the Advisory Committee on Immunization Practices (ACIP) recommended against LAIV use in 2016 to 2017 [12]; however, in 2018 to 2019, after presentation of new data, the ACIP included LAIV as a vaccine choice without a preferential recommendation [13]. Our study was planned before these conflicting data were known, but adds to the evidence base on our knowledge of pediatric influenza vaccines, including the potential for indigenously produced LAIV to be used for prevention of influenza virus infection in India. We conducted a 2-year, triple-blind, randomized controlled trial to determine the safety and absolute efficacy of LAIV and IIV and relative efficacy of LAIV versus IIV among children aged 2 to 10 years in rural India.

## Methods

### Study design and participants

The study was conducted using an existing surveillance platform established in June 2015 for acute respiratory tract infections among children in 6 villages of the Ballabgarh block (Faridabad district) in the northern state of Haryana, India [14,15]. All children aged 2 to 10 years registered in the demographic database of these villages were eligible (see S1 Table).

The trial consisted of 2 intervention arms, nasally administered LAIV or intramuscular IIV, and 2 control arms, intranasal placebo as LAIV control or inactivated polio vaccine (IPV) as IIV control. Although the trial was initially planned for 1 year, owing to the mild influenza season during the first year, it was extended for a second year to boost enrollment of influenza cases. The cohort was revaccinated during the second year per their original allocation, maintaining blinding of vaccinators, observers, and all study investigators throughout the study period (see S1 CONSORT Checklist).

### Recruitment, randomization, and masking

Children participating in the existing surveillance platform were medically examined by study physicians and excluded if they had any conditions that would make them ineligible (see S1 Text). Written informed consent from parents or guardians and written assent from children aged ≥7 years were obtained and audiovisually recorded for each year of the trial [16]. The study was approved by the Institutional Ethics Committee (IEC) of the All India Institute of India of Medical Sciences (AIIMS), New Delhi; Drug Controller General of India (DCGI), New Delhi; and Institutional Review Board of the Centers for Disease Control and Prevention (CDC), Atlanta, Georgia.

Individual randomization was conducted in blocks of 30 individuals with an allocation ratio of 2:2:1:1 for LAIV, IIV, intranasal placebo, and IPV, respectively. The random sequence was generated by a statistician not involved in the trial and concealed using sequentially numbered opaque sealed envelopes. Randomization keys were maintained in a separate, secure password-protected server. Vaccines were masked by the pharmacist and labeled with the appropriate administration route and a randomization code.

Children eligible for inclusion were invited to a community-based vaccination session organized at easily accessible community areas like schools or other locations. Children with nasal congestion or acute illness (e.g., temperature ≥37.5°C) at the time of vaccination were requested to come to the next session. Children were allocated to a specific arm per the random sequence by field investigators at the sessions and given masked vaccine or placebo by the appropriate route of administration.

### Procedures

All vaccines or placebo were administered from June 16 to June 30, 2015 (for Year 1) and from June 14 to July 14, 2016 (for Year 2). The influenza vaccination was done during the months of June before the onset of monsoons in the study [17,18] with the most recent formulation of available influenza vaccine as per WHO recommendations for the tropical countries. For Year 1, a second dose was required for children under 9 years of age in the IIV/IPV arms. These were administered at vaccination sessions held 4 weeks (July 14 to August 7, 2015) after the first dose sessions; if missed, follow-up home visits were conducted to reach unvaccinated participants. In Year 2, all children were administered only 1 dose of vaccine or placebo per their original allocation. In the LAIV/intranasal placebo arms, all children received one 0.5-ml dose equally divided and administered into both nostrils. In the IIV/IPV arms, in addition to the second dose requirement in Year 1, dose volume varied by age: children aged <3 years received either 0.25 ml IIV or 0.5 ml IPV and children 3 years and older received 0.5 ml of IIV or IPV. Composition of trivalent LAIV and IIV was per WHO southern hemisphere vaccine recommendation for the corresponding years. Thus, in Year 1, vaccines included influenza A/California/7/2009 (A(H1N1)pdm09-like), A/Switzerland/9715293/2013 (A(H3N2)-like), and influenza B/Phuket/3073/2013 –Yamagata-like viruses [19], and in Year 2, vaccines included influenza A/California/7/2009(H1N1)pdm09-like, influenza A/Hong Kong/4801/2014 (A(H3N2)-like), and influenza B/Brisbane/ 60/2008 –Victoria-like viruses [19]. The LAIV vaccine and similarly packaged intranasal placebo were provided by SII, and IIV and IPV were purchased from Sanofi Pasteur (India). Vaccine cold chain was maintained from the time of manufacturer transport to the field site and during vaccination sessions; temperature was monitored by logging thermometer readings.

All children were observed for 30 minutes after each vaccination and visited at home on days 1, 3, 7, 14, 21, 28, and 42 to monitor adverse events. Surveillance for serious adverse events (SAEs) defined as events resulting in death, hospitalization, immediately life-threatening conditions, or persistent or significant disabilities continued throughout the 2-year study period. The principal investigator (AK) performed the initial causality assessment on all reported SAEs and reported them to the IEC subcommittee for SAEs, which reviewed and forwarded them to the office of DCGI for a final decision on whether the SAE was attributable to a trial vaccine.

We conducted febrile acute respiratory infection (FARI) surveillance among enrolled children through weekly home visits throughout the 2 study years, which were defined as June 23, 2015 to June 30, 2016 (Year 1) and July 1, 2016 to June 27, 2017 (Year 2). FARI was defined as presence of measured (>38°C) or reported fever plus any of the following symptoms: cough, difficulty breathing, sore throat, nasal discharge, or earache/discharge. Surveillance was conducted by trained field investigators through interview of close caregivers, usually mothers. Study nurses performed a detailed clinical examination and collected nasal and throat swabs from children with FARI using Dacron swabs. Combined swabs were transported in viral transport media under cold chain to the field office on the same day as collection where they were stored at −70°C. On the following day, specimens were transported under cold chain to the virology laboratory at AIIMS, New Delhi for storage and testing.

In addition, baseline characteristics (e.g., age, sex, basic health, and socioeconomic indicators) of enrolled children were retrieved from census data collected earlier that year as part of ongoing demographic surveillance. Additional information on household-level factors, such as wealth index, fuel use, and household smoking, and individual factors, such as nutritional status of children, were collected through in-person interviews at the beginning of Year 2 using a standardized, pretested questionnaire. All data were entered into Epi Info version 7.0.

### Laboratory methods

Nasal and throat swab specimens were tested for influenza viruses using real-time reverse transcriptase polymerase chain reaction (rRT-PCR). Specimens were first tested for any influenza A and/or B viruses; subsequent assays identified influenza A subtype and B lineage [20]. For further genetic characterization, we tried to sequence as many clinical specimens (not from isolate) as possible ensuring inclusion of all influenza type/subtype viruses from all months of the study period at the National Institute of Virology (NIV), Pune using methods described in S2 Text.

### Outcomes

The primary efficacy end point was any laboratory-confirmed influenza virus infection among children aged 2 to 10 years with FARI beginning 14 days after vaccination. The secondary efficacy end points were laboratory-confirmed influenza by age group, by influenza type/subtype/lineage, and time to infection (to evaluate waning protection during the year). All primary and secondary end points were prespecified in the study protocol. Safety end points were solicited and unsolicited adverse events including SAEs, in the first 30 minutes postvaccination and during 42 days following receipt of any study vaccine or medically significant recurrent wheezing defined as physician-confirmed wheezing that occurred at least 2 times in the 12 months following receipt of any study vaccine.

### Statistical analyses

The study had 3 co-primary objectives: (1) to evaluate the relative efficacy of LAIV versus IIV and to evaluate the absolute efficacy of both (2) LAIV and (3) IIV versus a control vaccine to reduce the rate of laboratory-confirmed influenza virus infection among children with FARI aged 2 to 10 years in rural India. To estimate relative efficacy, the required sample size was 1,000 children each in the LAIV and IIV groups assuming 50% VE for IIV and 75% for LAIV [21], influenza virus attack rate of 10%, 80% power, alpha of 0.05, and 10% attrition. To estimate absolute efficacy of LAIV and IIV, controls were combined to include 500 children each from the IPV and intranasal placebo groups. We followed a modified intention-to-treat (mITT) analytic plan, defined a priori to include children per their original allocated arm, with censoring of children lost to follow-up. Tables were finalized using dummy allocation of subjects for both years of data before un-blinding the allocation codes at the end of the second year.

VE was calculated as 1 − hazard ratio (HR) × 100, where absolute efficacy of LAIV and IIV were calculated as the HR between LAIV or IIV and the control group, and relative efficacy was calculated as the HR between LAIV and IIV groups. VE was estimated for each study year using a Cox proportional hazards model (PH) [22] to account for seasonal variability in the risk of influenza virus infection and other time-varying covariates. Due to multiple study participants from same household, we also included random effect term for household (see S1 Fig). Observation period for each child began 14 days postreceipt of final vaccine dose each year. Children with at least 4 weeks of follow-up during the observation period for a given study year were included in the analysis until the earliest of the following censoring events: influenza virus infection (censored by virus type/subtype), last follow-up visit of that study year, or end of observation period.

As VE can vary widely by age, influenza type/subtype, and other factors, we conducted several secondary analyses. These included VE by age group (Year 1: aged <3 years, 3 to 4 years, 5 to 8 years, and 9 to 10 years and Year 2: aged 3 to 4 years, 5 to 8 years, and 9 to 11 years); influenza type/subtype; and time since vaccination. “Vaccine-matched subtype/lineage” was defined as virus detected by rRT-PCR among study participants that matched one of the 3 vaccine subtypes/lineages; and phylogenetic matching was defined at the strain level based on the sequence data performed on the subsample of influenza–positive specimens (see S2 Text).

We described the proportion of participants in each of the 4 study groups who reported adverse events of any severity (mild, moderate, or severe). Person-time was calculated in child-years. All analyses were done in Stata version 15.0. The trial was registered in the Clinical Trial Registry of India (Clinical Trial Registry No: CTRI/2015/06/005902).

## Results

**Fig 1:**
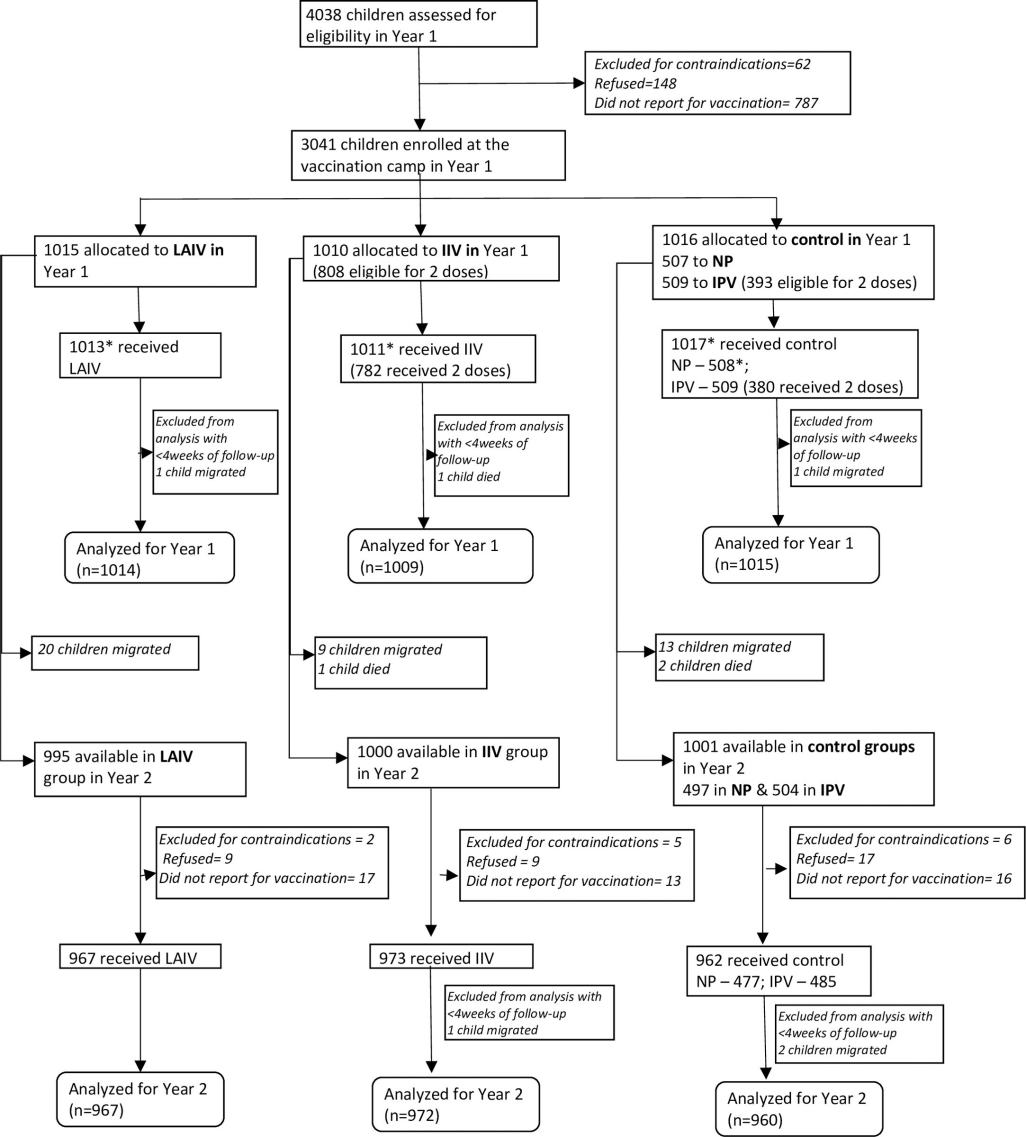
Flowchart of enrollment and follow-up in the trial. *Note: One child allocated to LAIV received IPV and one child received NP. IIV, inactivated influenza vaccine; IPV, inactivated polio vaccine; LAIV, live attenuated influenza vaccine; NP, nasal placebo.

For Year 1, 4,038 children were assessed for eligibility; 3.7% declined participation, and 1.5% had contraindications. Thus, 3,828 (94.8%) eligible children were consented and invited to vaccination sessions; of these, 3,041 (79.4%) reported to sessions and were allocated to LAIV (*n* = 1,015), IIV (*n* = 1,010), nasal placebo (*n* = 507), or IPV (*n* = 509) (Fig 1). During Year 1, 42 children emigrated and 3 died. For Year 2, of 2,996 (98.5%) eligible children, 2,915 (97.3%) reported to vaccination sessions, but 13 were excluded due to contraindications during reassessment. Thus, in Year 2, 2,902 (95.4% of 3,041 from Year 1) children received LAIV (*n* = 967), IIV (*n* = 973), nasal placebo (*n* = 477), or IPV (*n* = 485).

A total of 2,956 child-years of surveillance occurred in Year 1 and 2,775 child-years in Year 2. Among 5,731 child-years, 1,908 were among the LAIV arm, 1,916 were among the IIV arm, and 1,907 were among the control arm (see S2 Fig). Baseline characteristics including age, nutritional status, and household characteristics were similar between the 4 groups (Table 1). Overall, mean age was 6.5 years and 20% were aged 9 to 10 years; 23.3% to 29.6% of children were moderate to severely underweight; 79.4% to 85.2% of households used solid cooking fuel; and 53.9% to 57.1% of households had a smoker within the home. Among all 4 groups, mean household size was 7, with an average of 4 children aged <11 years.

**Table 1:** Baseline demographic characteristics of study participants by vaccination group.

Characteristics	LAIV (*n* = 1,015)	IIV (*n* = 1,010)	NP (*n* = 507)	IPV (*n* = 509)
**Mean (SD) age in years at enrollment**	6.50 (2.6)	6.55 (2.5)	6.39 (2.5)	6.62 (2.6)
Age group *n* (%)				
2–4 years	366 (36.1)	326 (32.3)	174 (34.3)	166 (32.6)
5–8 years	437 (43.1)	478 (47.3)	242 (47.7)	227 (44.6)
9–10 years	212 (20.9)	206 (20.4)	91 (18.0)	116 (22.8)
Proportion of female subjects *n* (%)	467 (46.1)	489 (48.4)	221 (43.6)	239 (47.0)
**Nutritional status**^a^	***n* = 968**	***n* = 985**	***n* = 490**	***n* = 496**
Stunted^b^				
None	408 (42.2)	448 (45.5)	199 (40.6)	194 (39.1)
Mild	340 (35.1)	326 (33.1)	178 (36.3)	173 (34.9)
Moderate or severe	220 (22.7)	211 (21.4)	113 (23.1)	129 (26.0)
Wasted^c^				
None	481 (49.7)	514 (52.2)	259 (52.9)	259 (52.2)
Mild	337 (34.8)	342 (34.7)	160 (32.7)	158 (31.9)
Moderate or severe	150 (15.5)	129 (13.1)	71 (14.5)	79 (15.9)
Underweight^c^	*n* = 702	*n* = 727	*n* = 365	*n* = 345
None	234 (33.3)	266 (36.6)	127 (34.8)	122 (35.4)
Mild	277 (39.5)	287 (39.5)	153 (41.9)	121 (35.1)
Moderate or severe	191 (27.2)	174 (23.9)	85 (23.3)	102 (29.6)
**Household characteristics of children (*n* = 2,764)**^d^	**(*n* = 913)**	**(*n* = 926)**	**(*n* = 463)**	**(*n* = 462)**
Number of households	501	504	336	318
Children living in households				
Using solid fuel *n* (%)	765 (83.8)	789 (85.2)	379 (81.9)	367 (79.4)
With smoker *n* (%)	492 (53.9)	529 (57.1)	257 (55.5)	261 (56.5)
With soap at toilet’s hand washing facility *n* (%)	457 (50.1)	476 (51.4)	243 (52.5)	252 (54.6)
Wealth quintile of study participants				
First (poorest)	176 (19.3)	159 (17.2)	76 (16.4)	93 (20.1)
Second	184 (20.2)	202 (21.8)	90 (19.4)	97 (21.0)
Third	177 (19.4)	177 (19.1)	92 (19.9)	92 (19.9)
Fourth	200 (21.9)	194 (21.0)	98 (21.2)	88 (19.1)
Fifth (richest)	176 (19.3)	194 (21.0)	107 (23.1)	92 (19.9)
Mean (SD) number of persons living in the household	7.0 (3.2)	7.1 (3.3)	6.9 (3.0)	6.9 (3.0)
Mean (SD) number of children <11 years in the household	2.8 (1.4)	2.8 (1.4)	2.8 (1.4)	2.7 (1.3)

^a^Data collected for 2,939 participant children.

^b^Z score, mild (–2 to <–1), moderate (–3 to <–2), or severe (<–3) by height for age. A stunted child may also be underweight or wasted.

^c^Z score for weight for height/body mass index calculated by WHO AnthroPlus. WHO AnthroPlus does not give Z score of weight for age for children above 10 years of age (thus not calculated for 800 children aged 10+ in the second year of study).

^d^Same households can be in more than 1 column.

IIV, inactivated influenza vaccine; IPV, inactivated polio vaccine; LAIV, live attenuated influenza vaccine; NP, nasal placebo; SD, standard deviation.

**Fig 2:**
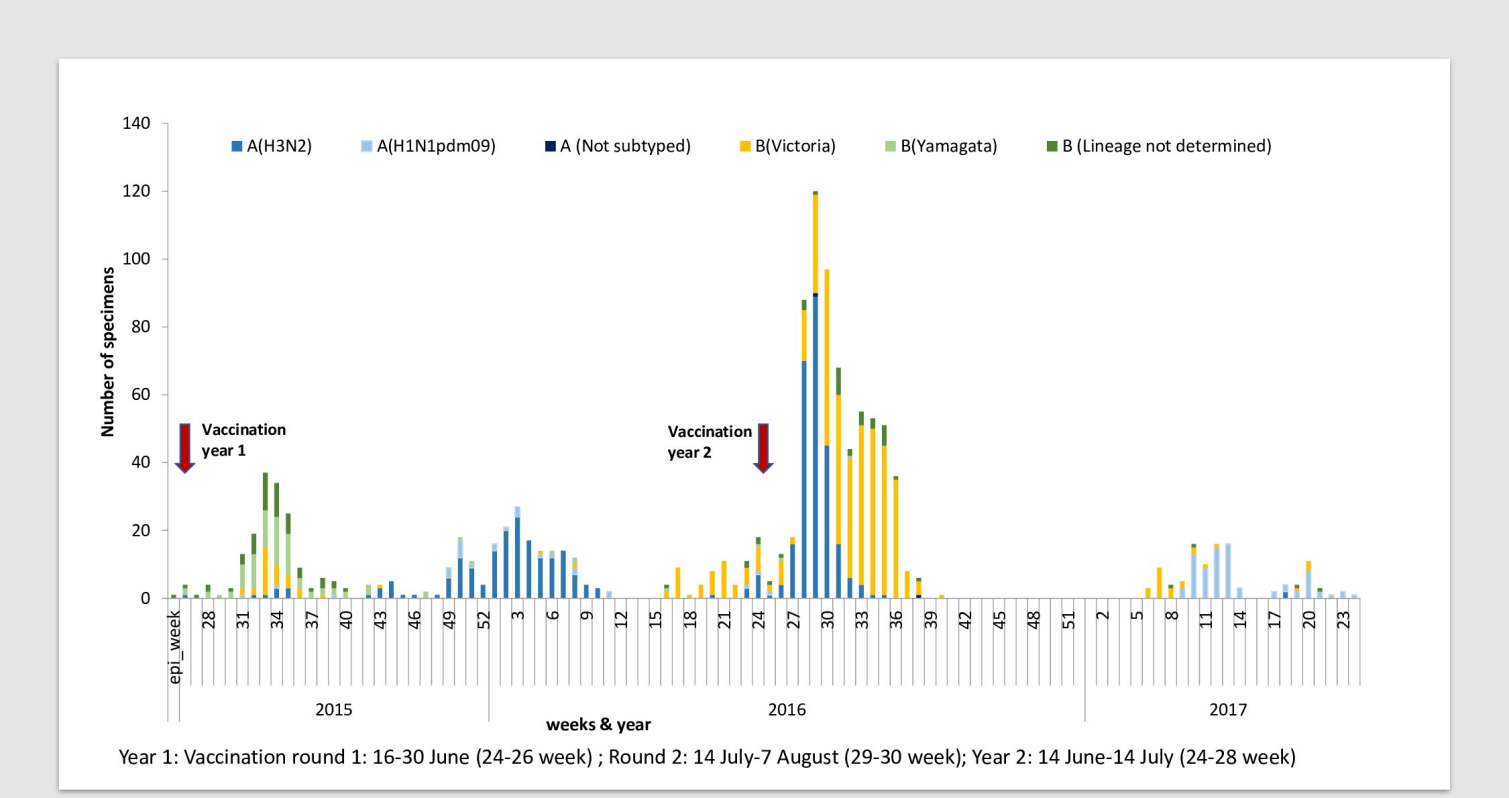
Influenza circulation in the study area during June 2015 to June 2017. Year 1: Vaccination round 1: June 16 to 30 (24–26 weeks); Round 2: July 14 to August 7 (29–30 weeks); Year 2: June 14 to July 14 (24 to 28 weeks).

During Year 1, influenza virus circulation peaked twice, in August 2015 (weeks 31 to 36, predominantly influenza B (Yamagata)) and from December 2015 to February 2016 (weeks 49 to 8, predominantly influenza A(H3N2)) (Fig 2). During Year 2, total influenza virus circulation was higher than in Year 1, with longer peak periods, from June 2016 through September 2016 (weeks 23 to 37, predominantly influenza B(Victoria) and A(H3N2)), followed by a smaller peak from February to March 2017 (weeks 7 to 13, predominantly influenza A(H1N1)pdm09).

During the study period, 19,981 FARI episodes were identified, out of which specimens could be collected and tested from 18,653 (93.4%) of specimens (Year 1: 9,285; Year 2: 9,368). In Year 1, of 3,041 enrolled children, 408 (13.4%) reported 435 influenza-associated FARI episodes (25 children had >1 influenza virus infection). In Year 2, of 2,902 children, 650 (22.4%) reported 747 influenza-associated FARI episodes (84 children had >1 influenza virus infection). After excluding influenza-associated FARI episodes that developed within 14 days of vaccination (*n* = 8) or before receipt of second dose of IIV or IPV (*n* = 4), there were 396 and 629 children with influenza virus infections in Year 1 and Year 2, respectively.

Among 1,182 positive specimens, 245 were genetically characterized at NIV: 23 of 96 A(H1N1)pdm09, 103 of 407 A(H3N2), and 119 of 527 B viruses (see S3 Fig).

Sequencing of the haemagglutinin (HA) genes of the first year A(H1N1)pdm09 viruses (*n* = 4) indicated that most of the viruses belonged to genetic clade 6B and were similar to 2015-2016 WHO Northern Hemisphere Vaccine Strain A/California/07/2009-like viruses [19]. The second year A(H1N1)pdm09 stains (*n* = 19) belonged to genetic clade 6B.1 and were genetically similar to clade 6B.1 A/Michigan/45/2015.

The majority (29/43) sequences of A(H3N2) (*n* = 43) belonged to 3C.2a clade of reference virus A/Hong Kong/4801/2014, and the remaining 14 viruses belonged to A/Singapore/INFIMH-16-0019/2016 virus, from subclade 3C.2a1. In Year 2, all 60 sequences were of genetic clade 3C.2a1. In Year 1 of the study, both lineages of Influenza B viruses i.e. B/Victoria/2/87 and the B/Yamagata/16/88 lineages co-circulated. All the 24 selected Yamagata lineage virus specimens belonged to genetic clade 3 of reference virus B/Phuket/3073/2013 viruses. All the 18 specimens of B/Victoria/2/87 lineage viruses belonged to genetic clade 1A. In Year 2, all the 77 type B viruses were of B/Victoria/2/87 lineage and belonged to clade 1A of reference B/Colorado/06/2017-like viruses and had 2 amino acid deletion in HA (amino acids 162 and 163) gene.

In Year 1, influenza-associated FARI was detected in 120/1,014 (11.8%) of LAIV recipients and 193/1,015 (19%) of control recipients, for an absolute VE against any influenza-associated FARI of 40% (95% CI 25.2 to 51.9) (Table 2). In Year 2, influenza-associated FARI was detected in 159/967 (16.4%) of LAIV recipients and 303/960 (31.6%) of control recipients, for an absolute VE against any influenza-associated FARI of 51.9% (95% CI 42.0 to 60.1) (Table 2). In Year 1, absolute VE of IIV was 59.0% (95% CI 47.8 to 67.9) as compared with 49.9% (95% CI 39.2 to 58.7) in Year 2. In Year 1, LAIV was significantly less efficacious than IIV (VE −46.2%; 95% CI −88.9 to −13.1) but had similar efficacy in Year 2 (VE of 4.2%; 95% CI −19.9 to 23.5).

**Table 2:** Absolute and relative VE of live attenuated and inactivated influenza vaccines in preventing laboratory-confirmed influenza illness, by study year.

	No. of laboratory-confirmed influenza cases/total (%)			Adjusted VE^a^ % (95% CI)
	LAIV	IIV	Control	
LAIV versus control (absolute VE)				
Year 1	120/1,014 (11.8%)	-	193/1,015 (19%)	40.0 (25.2 to 51.9)
Year 2	159/967 (16.4%)	-	303/960 (31.6%)	51.9 (42.0 to 60.1)
IIV versus control (absolute VE)				
Year 1	-	83/1,009 (8.2%)	193/1,015 (19%)	59.0 (47.8 to 67.9)
Year 2	-	167/972 (17.2%)	303/960 (31.6%)	49.9 (39.2 to 58.7)
LAIV versus IIV (relative VE)				
Year 1	120/1,014 (11.8%)	83/1,009 (8.2%)	-	−46.2 (−88.9 to −13.1)
Year 2	159/967 (16.4%)	167/972 (17.2%)	-	4.2 (−19.9 to 23.5)

^a^Estimated using Cox proportional hazards model, with household-level random effect.

CI, confidence interval; IIV, inactivated influenza vaccine; LAIV, live attenuated influenza vaccine; VE, vaccine efficacy.

**Fig 3:**
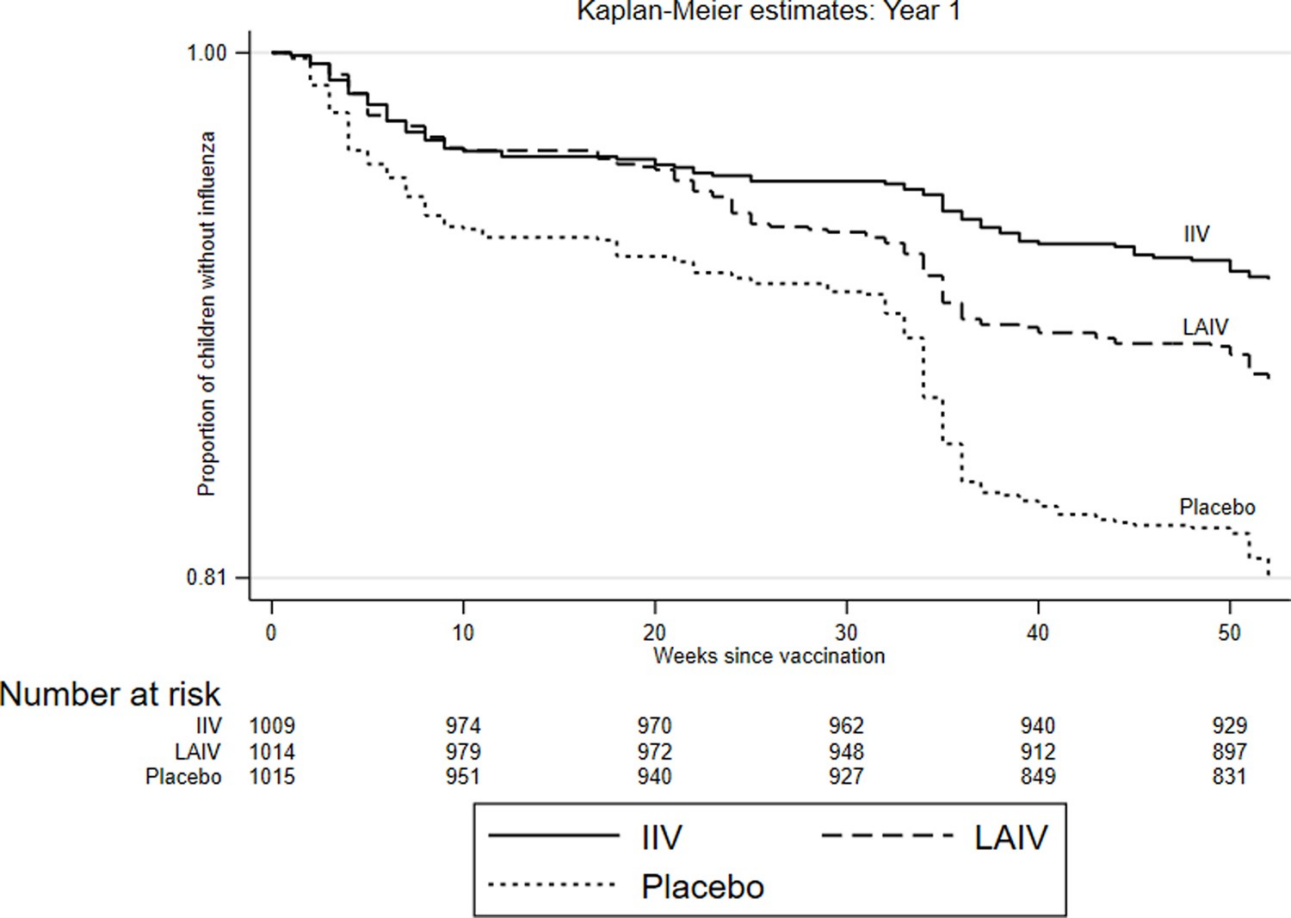
Kaplan–Meier curves of time to influenza infection by vaccine/placebo group for study Year 1. IIV, inactivated influenza vaccine; LAIV, live attenuated influenza vaccine.

**Fig 4:**
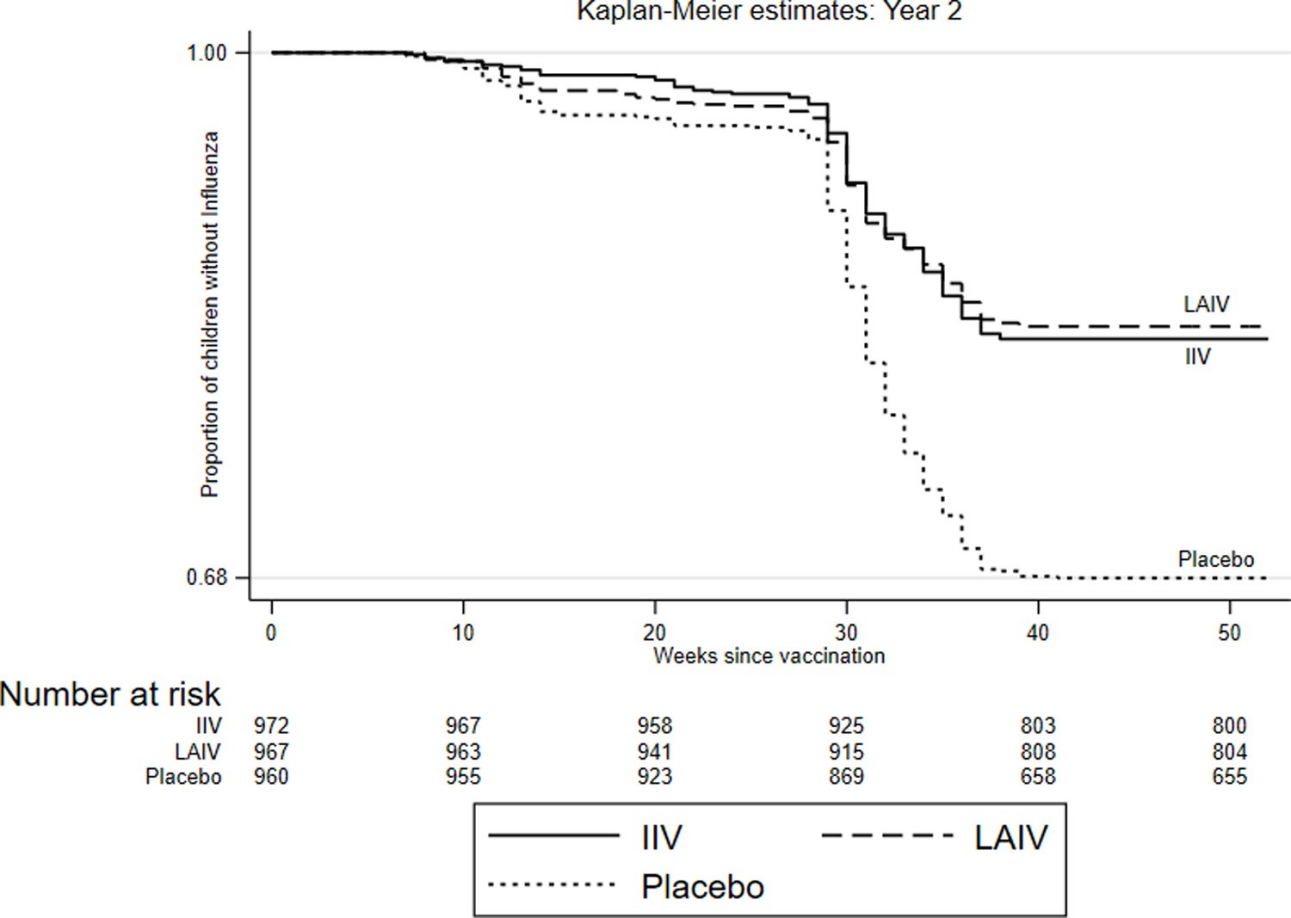
Kaplan–Meier curves of time to influenza infection by vaccine/placebo group for study Year 2. IIV, inactivated influenza vaccine; LAIV, live attenuated influenza vaccine.

In Year 1, IIV had the highest VE (72.0%) among children aged 5 to 8 years, whereas LAIV VE was 33.7% in this age group, for a relative efficacy of LAIV of −136.4% (95% CI −267.5 to −52.1) in this age group (Table 3). In the other 2 age groups, LAIV had similar efficacy to IIV in Year 1 and higher efficacy in Year 2, although these differences were not significant. While evaluating the time to infection (Figs 3 and 4), we observed that in the first 6 months postvaccination in both years, relative efficacy of LAIV compared with IIV was not significantly different (Year 1 (−24.0%; 95% CI −79.7 to 14.5) and Year 2 (10.6%; 95% CI −14.1 to 29.9)). In the second half of the year postvaccination, LAIV performed poorly especially in Year 1 with relative efficacy compared with IIV of −75.4 (95% CI −162.4 to 17.3).

**Table 3:** Absolute and relative VE of LAIV and IIV in preventing laboratory-confirmed influenza illness, by age group and influenza subtype/lineage.

Subgroups	Total number of infections (LAIV/IIV/Control)						Adjusted VE^a^ % (95% CI)					
							LAIV versus Control		IIV versus Control		LAIV versus IIV	
	Year 1			Year 2			Year 1	Year 2	Year 1	Year 2	Year 1	Year 2
Age group (Years)												
2–4 (Year 1)	48	41	75	47	56	90	43.8	57.2	45.6	46.9	−3.5	20.5
3–4 (Year 2)							(20.1 to 60.5)	(39.9 to 69.6)	(21.6 to 62.3)	(27.0 to 61.4)	(−53.9 to 30.4)	(−17.0 to 45.9)
5–8	58	28	91	86	70	154	33.7	46.7	72	58.9	−136.4	−29.3
							(6.6 to 52.9)	(30.9 to 58.9)	(56.0 to 82.2)	(45.2 to 69.2)	(−267.5 to −52.1)	(−77.9 to 6.1)
9–10 (Year 1)	14	14	27	26	41	59	50.4	59	48.9	33.6	2.9	37.7
9–11 (Year 2)							(11.0 to 72.4)	(33.9 to 74.6)	(3.7 to 72.9)	(2.1 to 55.0)	(−101.7 to 53.2)	(−5.2 to 63.1)
Virus type and subtype/lineage												
A(H1N1)pdm09	12	8	5	24	15	32	−140.2	25.9	−60.9	54.2	−49.4	−61.9
							(−580.4 to 15.2)	(−21.1 to 54.6)	(−390 to 47.2)	(16.0 to 75.1)	(−265.0 to 38·9)	(−181.6 to 6.9)
A(H3N2)	47	43	91	57	60	109	49.4	49	53.5	46.6	−9	4.6
							(29.1 to 63.8)	(28.3 to 63.7)	(34.6 to 67.0)	(26.0 to 61.4)	(−63.9 to 27.5)	(−38.0 to 34.1)
B(Yamagata)	16	11	53	1	1	0	70.2	-	79.5	-	−45.3	-
							(50.0 to 82.3)	-	(62.0 to 89.0)	-	(−202.6 to 30.2)	-
B(Victoria)	38	17	27	88	92	183	−41.2	55	37	53	−124.6	4.3
							(−129.6 to 13.1)	(42.6 to 64.7)	(−15.4 to 65.7)	(38.5 to 64.1)	(−279.2 to −33.0)	(−29.1 to 29.0)
All viruses in trivalent vaccine^b^	73	59	144	156	157	293	51	51	60.4	51.3	−23.6	−0.3
							(35.7 to 62.7)	(40.9 to 59.4)	(47.8 to 69.9)	(40.3 to 60.2)	(−70.9 to 10.6)	(−26.0 to 20.1)
Time since vaccination												
Less than 6 months	59	48	131	130	145	268	56.6	55.4	65	50.2	−24	10.6
							(40.7 to 68.2)	(45.1 to 63.7)	(52.6 to 74.1)	(38.5 to 59.6)	(−79.7 to 14.5)	(−14.1 to 29.9)
Greater than 6 months	61	35	62	29	22	35	2.7	18.1	43.8	38.5	−75.4	−33.2
							(−36.7 to 30.8)	(−30.4 to 48.5)	(15.7 to 62.6)	(−3.2 to 63.4)	(−162.4 to 17.3)	(−122.5 to −20.2)

^a^Estimated using Cox proportional hazards model, with household-level random effect.

^b^Includes all confirmed influenza infections contained in trivalent vaccine for a given study year (Year 1 –A(H1N1)pdm09, A(H3N2), B(Yamagata); Year 2 –A(H1N1)pdm09, A(H3N2), B(Victoria)).

CI, confidence interval; IIV, inactivated influenza vaccine; LAIV, live attenuated influenza vaccine; VE, vaccine efficacy.

Local and systemic reactions were uncommon and similar between LAIV, IIV, nasal placebo, and IPV groups (Table 4). Local adverse events were more common in the injection group/s as compared with the nasally administered groups. Recurrent wheezing was reported by approximately 1% of children over the 2-year period and not significantly different between groups. SAEs were rare, and none were attributed to be causally related to vaccination.

**Table 4:** Adverse events associated with live attenuated and inactivated influenza vaccines within 42 days post-administration, aggregated across study years.

Adverse events	LAIV (*n* = 1,978)^a^		NP (*n* = 986)^a^		IIV (*n* = 2,766)^a^		IPV (*n* = 1,374)^a^	
SAEs^b^	3		8		5		1	
SAEs attributed to vaccine	0		0		0		0	
Medically significant recurrent wheezing	32 (1.6)		13 (1.3)		29 (1.0)		20 (1.4)	
Any local adverse event^c^	223 (11.3)		94 (9.5)		958 (34.6)		452 (32.9)	
Systemic adverse events	Mild	Moderate to severe	Mild	Moderate to severe	Mild	Moderate to severe	Mild	Moderate to severe
Fever	410 (20.7)	106 (5.4)	208 (21.1)	69 (7.0)	635 (23.0)	195 (7.0)	328 (23.9)	121 (8.8)
Headache	85 (4.3)	27 (1.4)	42 (4.3)	22 (2.2)	144 (5.2)	57 (2.1)	81 (5.9)	29 (2.1)
Diarrhea	53 (2.7)	90 (4.6)	17 (1.7)	44 (4.5)	78 (2.8)	127 (4.6)	40 (2.9)	78 (5.7)
Vomiting	75 (3.8)	43 (2.2)	35 (3.5)	22 (2.2)	115 (4.2)	71 (2.6)	53 (3.9)	40 (2.9)
Decreased feeding	83 (4.2)	5 (0.3)	47 (4.8)	4 (0.4)	156 (5.6)	14 (0.5)	69 (5.0)	5 (0.4)
Lethargy	61 (3.1)	2 (0.1)	37 (3.8)	2 (0.2)	117 (4.2)	8 (0.3)	61 (4.4)	1 (0.1)
Irritability/excessive crying	29 (1.5)	5 (0.3)	19 (1.9)	2 (0.2)	57 (2.1)	9 (0.3)	38 (2.8)	2 (0.1)

^a^Represents total number of doses.

^b^Defined as events which resulted in death, were life-threatening, required hospitalization, or lengthened hospitalization, resulting in persistent or significant disability.

^c^Local adverse event solicited were stuffy nose for LAIV and placebo and tenderness at injection site and redness at injection site and swelling at injection site for IIV and IPV.

IIV, inactivated influenza vaccine; LAIV, live attenuated influenza vaccine; NP, nasal placebo; SAE, serious adverse event; VE, vaccine efficacy.

## Discussion

This 2-year, triple-blind, large community-based trial demonstrated that both indigenously produced Russian-backbone LAIV and commercially available IIV were safe and moderately efficacious against laboratory-confirmed influenza-associated FARI among children aged 2 to 10 years in rural India. In Year 1, single-dose LAIV provided some protection to a vaccine-naïve population but significantly less than IIV, whereas in Year 2, LAIV and IIV efficacies were similar. Importantly, during both years, both vaccines were protective against influenza A(H3N2) viruses but had limited efficacy against influenza A(H1N1)pdm09 which was vaccine-matched but had low incidence. Both vaccines were protective against vaccine-matched influenza B viruses but not mismatched B viruses [19].

Although the absolute efficacy of both LAIV and IIV was moderate and ranged between 40.0% and 59.0% in both years, the relative efficacy of LAIV versus IIV, which was the primary outcome of interest, was poor in Year 1 (−46.2%) but improved (4.2%) in Year 2 (Table 2). The reasons for this are likely multifactorial and include the fact that only a single dose of SII Russian-backbone LAIV was required per the manufacturer’s instructions, whereas other LAIVs require 2 first doses for full effect[12]. An earlier meta-analysis by Ambrose and colleagues had shown higher relative efficacy of LAIV against IIV among children aged 6 months to 18 years across 4 studies conducted during years 2002 to 2005, although the vaccine dosages were different. A probable reason could be the absence of H1N1pdm09 strain during those years as other studies have also shown that LAIV had poor VE against H1N1pdm09 strain [10]. In addition, because LAIV is administered nasally, it is possible that some vaccine was wasted due to crying or overflow; thus, some children may not have received a full dose. Further nasal shedding analyses from this trial may further our understanding of LAIV virus uptake in young children in rural Indian settings with hot climates.

Our observed VE estimates were consistent with or higher than recent findings from other RCTs, for both LAIV and IIV. The 49.9% to 59.0% VE of IIV in this study was within the range of 26% to 74% as reported over 3 years from an earlier trial in India [5] and higher than that from an IIV study conducted from 2010 to 2014 in Bangladesh (VE 31%; 95% CI 18 to 42) [23]. LAIV VE (40.0% to 51.9% range) reported from this study is similar to that reported in the LAIV trial conducted in Bangladesh [6] (VE 41.0% (95% CI 28.0 to 51.6)) and much higher than that reported in the Senegal [9] (VE 0%; 95% CI −26.4% to 20.9%) trial. In our trial, LAIV was well matched to circulating influenza A viruses in both years, but A(H1N1)pdm09 was not the major circulating strain which may have limited our ability to estimate VE reliably for A(H1N1)pdm09. As we used trivalent vaccines in this study, we were able to observe VE for vaccine lineage or non-vaccine lineage influenza B viruses and did not see evidence of cross-protection against non-vaccine lineage (i.e., VE for influenza B/Victoria, in Year 1). The varying VE between the studies is multifactorial and likely due to heterogeneous study populations, differing conditions in each country and diversity of circulating influenza viruses, with some mismatch in each study but not for the same viruses. It has also been noticed that A/California/7/2009-like viruses in LAIV had lower immunogenicity compared to more recent A(H1N1) vaccine strains [24].

This study also looks at VE among school-aged children, while other trials (such as those in Senegal [9] and Bangladesh [6]) have typically targeted younger children, such as those 5 years and younger. While IIV and LAIV were similarly efficacious in children aged 2 to 4 years for both years of the study, we did observe significant differences between vaccine products among children 5 to 8 years during Year 1. This difference was not detected in Year 2 and may reflect a more robust response to the second dose of IIV in Year 1 in this older age group. The low relative VE of LAIV in this age group during Year 1 may also be attributed to trends in infection between age groups, both by subtype or over time within the year, but we were insufficiently powered to evaluate these factors.

In both years, efficacy was higher during the first 6 months postvaccination similar to previous studies conducted in this area [5], which was when influenza circulation peaked. Observational data from the US indicate that VE can decline within a single influenza season (“waning immunity”) [25]. In our study area, with biannual peaks and year-round circulation of influenza viruses, timing of vaccination and waning immunity may influence VE [18,26]. However, these trends are best understood by following VE over time by influenza virus subtype or lineage, which we did not have sufficient power to analyze.

Strengths of this study included 2 years of active surveillance over 2 different influenza seasons, high retention rates with adequate sample size to determine relative efficacy, and meticulous cold chain maintenance. Our study was also subject to several limitations. First, dosage requirements for vaccine-naïve children differed between products (single dose of LAIV versus 2 doses of IIV). This may have resulted in increased protection from those receiving a second dose of IIV, an important consideration in interpreting the relative VE during Year 1. Second, vaccines were administered in the summer months shortly before typical influenza season (monsoon season) [27], and the requirement of a second dose in the IIV arm reduced the postvaccination surveillance time in this arm, which began 2 weeks after receipt of final dose. Infections that occurred before this time point were not included in the analysis, but these were very infrequent and unlikely to influence absolute or relative efficacy of IIV. Third, we did not conduct immunogenicity studies, which could have helped explain some of our findings such as differences in VE by age groups or subtypes. Finally, our study was conducted in one rural site in India; thus, our results may not be generalizable to other settings.

Currently, influenza vaccines are not part of the national immunization programs in India; there are recommendations for their use among older adults and individuals with underlying conditions, but not among children [28]. Importantly, both IIV and LAIV were safe with moderate efficacy and performed better when there was vaccine matched to circulating influenza B lineage and adequate influenza prevalence particularly in the 6 months following vaccination. Given that VE for influenza B was adequate during lineage-matched seasons, quadrivalent vaccines regardless of route of administration should be considered as part of routine annual influenza immunization in India. As national and state-level public health programs in India consider an investment in influenza vaccination, both IIV and LAIV could be used to prevent pediatric influenza infection.

## Supporting information

S1 Table Protocol. (DOCX)

S1 CONSORT Checklist (DOCX)

S1 Text Exclusion criteria: list of conditions for exclusion of children from study. (DOCX)

S2 Text Sequencing procedures: procedure for sequencing of selected influenza–positive specimens. (DOCX)

S1 Fig Sample model output: model output for the primary end points. (TIF)

S2 Fig Analysis flowchart: description of data flow for mITT analysis of LAIV trial. LAIV, live attenuated influenza vaccine; mITT, modified intention-to-treat.
(TIF)

S3 Fig Phylogenetic analysis: phylogenetic analysis of influenza virus strains detected in Year 1 and Year 2. (TIF)

## Abbreviations

ACIP: Advisory Committee on Immunization Practices
AIIMS: All India Institute of India of Medical Sciences
CDC: Centers for Disease Control and Prevention
CI: confidence interval
DCGI: Drug Controller General of India
FARI: febrile acute respiratory infections
HA: haemagglutinin
HR: hazard ratio
IEC: Institutional Ethics Committee
IIV: inactivated influenza vaccine
IPV: inactivated polio vaccine
LAIV: live attenuated influenza vaccine
LMIC: low- and middle-income countries
mITT: modified intention-to-treat
NIV: National Institute of Virology
PH: Cox proportional hazards model
rRT-PCR: real-time reverse transcriptase polymerase chain reaction
SAE: serious adverse event
VE: vaccine efficacy
WHO: World Health Organization
