# Supplementary material for: Efficacy of live attenuated and inactivated influenza vaccines among children in rural India: A 2-year, randomized, triple-blind, placebo-controlled trial
Source: PLoS Med. 2021 Apr 29;18(4):e1003609. doi: 10.1371/journal.pmed.1003609 (PMC8118535; doi:10.1371/journal.pmed.1003609)
Supplement: S1 Table — (DOCX) [file pmed.1003609.s001.docx]

| **Title** | Assessment of the Efficacy of Licensed Seasonal Live Attenuated and Inactivated Influenza Vaccines among Children in India |
| --- | --- |
| **Study Population:** | Children 2-10 years of age residing in six villages in Ballabgarh Block, Haryana State, northern India |
| **Intervention**  **Arms:** | - One dose of 0.5ml of intranasal trivalent live attenuated influenza vaccine (LAIV) (Serum Institute of India, Pune. India) - One or two doses (age dependent) of intramuscular Trivalent inactivated influenza vaccine (IIV)(Sanofi Pasteur, Lyon France) - One or two doses (age dependent) of intramuscular Inactivated poliomyelitis vaccine (IPV) (Sanofi Pasteur, Lyon, France) - Intranasal placebo in same manner as LAIV(Serum Institute of India) |
| **Primary**  **Objectives:** | 1. To evaluate the total absolute efficacy of **live-attenuated influenza vaccine** in reducing laboratory-confirmed influenza symptomatic illness among children 2-10 years of age, as compared to a control vaccine (intranasal placebo or inactivated polio vaccine). 2. To evaluate the total relative efficacy of **live-attenuated influenza vaccine** in reducing laboratory-confirmed influenza symptomatic illness among children 2-10 years of age, as compared to inactivated influenza vaccine. 3. To evaluate the total absolute efficacy of **inactivated influenza vaccine** in reducing laboratory-confirmed influenza symptomatic illness among children 2-10 years of age, as compared to a control vaccine (intranasal placebo or inactivated polio vaccine). |
| **Secondary**  **Objectives:** | 1. To describe the safety profile of LAIV and IIV including immediate, local or systemic reactogenicity, and any systemic or serious adverse events among vaccinated children, as well as long-term wheezing outcomes. |
| **Methodology** | - This study would be carried out on an ARI surveillance platform which has been established in 6 villages within the Ballabgarh subdivision of Faridabad District, Haryana as part of an ongoing co-agreement with CDC, Atlanta. - The primary study outcome will be laboratory-confirmed clinical influenza infection for vaccine matched strains in vaccinated children, which will be identified through ongoing active surveillance for acute respiratory illnesses in the study population and detection of virus on respiratory swabs. Secondary outcomes will include clinical measures of safety, different strains and over time, and a comparison of efficacy of LAIV and IIV. - **Number of Patients**: 3300; (1100 each in two vaccine arms namely LAIV (Intranasal) and IIV (Intra muscular) and 550 each in two control arms (Intranasal Placebo and intramuscular IPV) - **Inclusion criteria**: Subjects must meet ALL of the following inclusion criteria before enrollment and randomization:  1. Aged 2-10 years 2. Resident of one of the study villages (“Resident” defined as sleeping in the house at least 6 months of the current calendar year) 3. Agrees to follow-up through weekly household visits 4. Parent/guardian provides informed consent 5. Children aged above 7 years will provide written assent and those below 7 years will provides verbal assent  - **Exclusion criteria**: Eligible Subjects will be excluded if they meet any of the following exclusion criteria:  1. Prior receipt of influenza vaccine for the study season 2. Any serious, active, medical conditions, including:    - chronic disease of any body system    - chronic infections such as tuberculosis    - genetic disorders, such as Down’s syndrome or other cytogenetic disorder    - known or suspected disease of the immune system    - Chronic or active wheezing illness 3. Received immunosuppressive agents including systemic corticosteroids during the month prior to study vaccination. 4. Less than 5 years who have a history of recurrent wheezing 5. Children receiving concomitant aspirin 6. History of Guillain-Barré Syndrome that occurred after receiving influenza vaccine in the past 7. History of allergy to chicken eggs or who are allergic to any of the vaccine ingredients like gelatin. 8. Current or past participation (within 2 months of trial enrollment visit) in any clinical trial involving any investigational products. 9. History of a previous severe allergic reaction with generalized urticaria, angioedema, or anaphylaxis. 10. Any condition determined by investigator as likely to interfere with evaluation of the vaccine or to be a significant potential health risk to the child or make it unlikely that the child would complete the study. 11. Administration of another live vaccine in the month prior to vaccination or for the month following vaccination.   **Temporary exclusion criteria:**   1. Nasal congestion that limits delivery of the vaccine to the nasal lining. 2. Acute illness accompanied by a body temperature of 37.5°C or above (axillary measurement) 3. Any acute respiratory infection within 14 days of enrollment visit.   Any illness accompanied by active wheezing within 14 days of enrollment visit.   - **Control(s)** : 1100 (550 each in the two control arms : Placebo (Intra nasal) and IPV(intra-muscular)) - **Study design** : Phase 4 placebo-controlled, triple-blinded, individual-randomized vaccine effectiveness trial of LAIV and IIV trivalent inactivated influenza vaccine . - **Dosages of drug**  \| **Vaccine** \| **All Enrollees*** \| \| **2-8 Years** \| **9-10 Years** \| \| --- \| --- \| --- \| --- \| --- \| \| **Dose** \| **Route** \| **No. of Doses** \| **No. of Doses** \| \| IIV \| 0.5# mL \| Intramuscular (right deltoid) \| 2 \| 1 \| \| IPV \| 0.5 mL \| Intramuscular \| 2 \| 1 \| \| LAIV \| 0.5 mL \| Intranasal \| 1 \| 1 \| \| Placebo \| 0.5 mL \| Intranasal \| 1 \| 1 \| \| *Children 2-8 years receiving LAIV or intranasal placebo require only one dose for “full vaccination”. Children 2-8 years receiving IIV or IPV will receive one dose of study vaccine at study entry, followed by a second dose one month later. All children 9-10 years of age will receive one dose only at study entry for all vaccines.  # Children 2-3 years will receive pediatric dosage of 0.25ml of IIV \| \| \| \| \|  - **Duration of Follow-up:** Each enrollee will be followed-up weekly through house visits by field workers for 12 months for assessment of acute respiratory disease or fever occurring in the previous seven days. Nasal and throat swabs will be taken from enrollees that meet the case definition. Also, nasal swabs will be collected from 150 enrollees across groups on day 2 and day 4 to test for shedding and vaccine uptake. - **Investigation specifically related to projects**  1. Throat swabs from FARI cases (New onset in last 7 days of reported or measured fever (>39C) plus one or more of the following: Cough, Sore Throat ( in those over 2 years), Ear-ache, Running Nose/Coryza, Rapid breathing / Shortness of breath in the one year follow up period post vaccination to look for influenza virus. These will be collected by trained nurses 2. All nasal swab and throat swab specimens will be tested for evidence of influenza virus using real-time RT-PCR (rRT-PCR) primers and probes for Influenza A and B and to further characterize into H3, pandemic H1, B (Victoria) and B (Yamagata). |
| Sample size calculations | For the primary study analysis of the absolute total efficacy of LAIV versus either intranasal or intramuscular placebo, we will assume a VE of 75% for LAIV, and an influenza attack rate of 10% in the study population. In order to detect a VE of 75% in effectiveness with a power of 80%, with 20% loss to follow-up, we will need to enroll n=200 into LAIV and placebo groups (n=100 into each arm of IPV and intranasal placebo).   \|  \| **Attack rate** \|  \| **VE** \| **Sample size** \| **Objective** \| \| --- \| --- \| --- \| --- \| --- \| --- \| \| **Placebo** \| **0.10** \|  \|  \| **164** \|  \| \| **LAIV** \| **0.025** \|  \| **0.75** \| **164** \| **Detect 75% VE** \| \|  \|  \|  \|  \| **328** \|  \|   For the primary study analysis of the relative effectiveness of LAIV versus IIV, we will assume a VE of 75% for LAIV and 50% for IIV, and an influenza attack rate of 10% in the study population. In order to detect an absolute difference of 25% in effectiveness with a power of 80%, with 20% loss to follow-up, we will enroll n=1100 into each arm of LAIV and IIV and control (n=550 into each arm of IPV and intranasal placebo).   \|  \| **Attack rate** \| **Relative risk** \| **VE** \| **Sample size** \| **Objective** \| \| --- \| --- \| --- \| --- \| --- \| --- \| \| **Placebo** \| **0.10** \|  \|  \| **906** \|  \| \| **LAIV** \| **0.025** \| **0.25** \| **0.75** \| **906** \| **Detect 25% VE difference** \| \| **IIV** \| **0.05** \| **0.5** \| **0.5** \| **906** \| \|  \|  \|  \|  \| **2718** \|  \| |
| **Study drug Acquisition** | All study vaccines will be procured from or donated by the manufacturer. We will use VAXIGRIP trivalent inactivated influenza vaccine and IMOVAX inactivated poliovirus vaccine, purchased from Sanofi Pasteur SA, Lyon, France. Live attenuated influenza Vaccine and Intra Nasal control would be provided free of cost by Serum Institute of India, Pune, India. |
| **Randomization & Blinding procedures** | This is a triple blinded study. The randomization schedule, prepared by an unblinded statistician who will not be involved in the data cleaning or analysis, will be maintained in a separate, secure location from the central study database, on a password protected server, to ensure that code is not broken prior to study termination.  This statistician would generate thirty alpha numeric codes (five digit each) for the four different agents being used in the study (i.e. ten each for the IIV and the LAIV and five each for the two controls). These codes would be used to make one randomization block (Thus each block would contain codes for these four agents in the ration of 2:1:2:1 for LAIV, intranasal placebo, IIV, and IPV). A total of 110 such blocks shall be generated (the codes of each block would remain the same but there sequence shall be generated randomly for each block). These will form the randomization list.  In the Delhi office the study vaccines will be masked by study pharmacist in Delhi office following acquisition. The statistician involved in randomization would share the 30 alpha numeric codes with the pharmacist. The pharmacist would generate 110 label pairs of the each code ( hence would have a total of 3300 label pairs would be generated). One of each of the label pair would be used to re label the vaccine after masking the original label. The appropriate administration route, would also be affixed. The unused label from each of the label pairs shall be put inside the packing for use in the field.  Subject randomization will occur when children enrolled in the study visit the vaccination camp. Children attending the camp would be sequentially assigned codes as per the random list. The person giving the vaccine would take out vaccine with the matching code and administer the same to the child. The extra label inside vaccine packing would also be put on the child record sheet as a double check for the correctness in matching of labels used in choosing the vaccine.  The randomization schedule will only be shared as needed and by written approval from study investigators once a statistical analysis plan has been determined. The schedule will also be shared with independent study monitors, as and when required for investigation of any adverse events. |
| **Safety measures** | Safety parameters will include the proportion of subjects experiencing specific symptoms falling into one of the following three categories:   - Immediate reactions (reactions within 30 minutes after injection, with emphasis on allergic reactions), measured by observation of symptoms/problems by study medical staff. - Solicited local and systemic reactions and other unsolicited adverse events occurring during the 28 days following receipt of any dose of vaccine, measured via diary card completed by parent/guardian following immunization. - Serious adverse events occurring within 12 months following receipt of any study vaccine and deemed to be at least possibly related to receipt of study vaccine, measured as observed by study staff and/or reported by parent/guardian at any time during routine surveillance. - Medically significant wheezing confirmed by study physicians that occurs at least two times (recurrent wheezing) in the 12 months following receipt of any study vaccine. - The **severity** of all adverse events will be classified as either mild, moderate, or severe by the investigator or a designated, qualified healthcare professional. The degree of incapacity caused by the adverse event and the level of medical intervention required for treatment may be helpful in assessing the overall severity of the adverse event. For example: - “**Mild**” events are generally regarded as noticeable but have no impact on normal activities; they may or may not require over-the-counter treatment managed by the subject. - “**Moderate**” events generally have some impact on an individual’s normal activities and may require general symptomatic medical intervention by a healthcare professional or by the subject. - “**Severe**” adverse events may be incapacitating, leading to suspension of normal daily activities, and would generally require more immediate medical evaluation and intervention by a healthcare professional. - For all adverse events, the investigator and the monitor will determine a causal relationship, to the study vaccine without knowledge of which study vaccine was administered. A number of factors will be considered in making this assessment, including: 1) the temporal relationship of the event to the administration of the study vaccine 2) whether an alternative etiology has been identified and 3) biological plausibility - Serious adverse events will be reported to the DSMB, DCGI, the IRBs/ECs of AIIMS and CDC Atlanta according to each institution’s regulations   The study vaccines are all licensed by DGCI for use in the study population. |
| **Study period** | The trial was initially planned for one year (June 2015-June 2016). However, owing to the mild influenza season during the first year, it was extended for a second year (July 2016- June 2017) to boost enrollment of influenza cases. After obtaining IEC clearance, the cohort was re-vaccinated during the second year per their original allocation, maintaining blinding of vaccinators, observers, and all study investigators throughout the study period. |
| **Clearance and Compliance** | - **Ethics Committee review**   The AIIMS Institutional ethical clearance has been granted on 12/12/2014 (year 1); 07/04/2016 (year 2).   - **Informed consent**   The process of consenting for each participant will be video graphed as per the current ethical guidelines for clinical trials in the country, In addition, verbal assent will be obtained and appropriately documented for children younger than 7 years, whereas written assent will be obtained for minor children 7 years and older.   - **Vulnerable populations:** *Children*   This study is designed specifically to include children 2-10 years of age to receive vaccinations. This population is at high risk for influenza infection, including severe disease, and is recommended as a target group for influenza vaccination by the World Health Organization. Evaluating the effects of IIV and LAIV in this age group is a key research question, as data suggest that LAIV may be more beneficial in this age group than in older age groups.   - All vaccines used in the trial have been licensed and recommended for use in the study population. - Adhering to regulatory compliance requirements, the study would be conducted in accordance with the International Conference on Harmonization-Good Clinical Practice (ICH-GCP) guidelines; the Declaration of Helsinki; Guidelines for Clinical Trials on Pharmaceutical Products in India – GCP Guidelines issued by the Central Drugs Standard Control Organization (CDSCO), 2001 and Ethical Guidelines for Biomedical Research on Human Subjects issued by the Indian Council of Medical Research (ICMR), 2006. |
| **Analyses:** | **Efficacy**  The total absolute efficacy of LAIV and IIV in reducing rates of laboratory-confirmed influenza for matched strains and the relative efficacy of LAIV over IIV will be calculated through Cox proportional hazards model to account for seasonal variability in risk of influenza and other time-varying covariates.  Vaccine efficacy will be measured as the reduction in rates of laboratory-confirmed influenza between arms and will be calculated as (1- RR_LAIV/IIV_) X 100, where RR_LAIV/IIV_ is the relative risk of laboratory-confirmed influenza in study participants vaccinated with LAIV versus those vaccinated with IIV.  Several secondary efficacy analyses will be conducted:  Efficacy of LAIV and IIV against antigenically drifted influenza viruses, defined as reduction in laboratory-confirmed influenza infection to non-vaccine strain influenza viruses.  Duration of protection by vaccination with LAIV and IIV against influenza viruses, defined as the efficacy of LAIV and IIV in reducing laboratory-confirmed influenza infection in terms of time since vaccination (less than six months and greater than six months)  **Safety**  The primary safety endpoints will be the total number of solicited and unsolicited adverse events in the 42 days following vaccination, categorized as “Mild”, “Moderate”, “Severe”, or “Any adverse event”, as well as the number of children in each vaccine group who report any severe adverse event or recurrent medically significant wheezing 12 months after vaccination. |
