# Supplementary material for: Efficacy of live attenuated and inactivated influenza vaccines among children in rural India: A 2-year, randomized, triple-blind, placebo-controlled trial
Source: PLoS Med. 2021 Apr 29;18(4):e1003609. doi: 10.1371/journal.pmed.1003609 (PMC8118535; doi:10.1371/journal.pmed.1003609)
Supplement: S1 Text — (DOCX) [file pmed.1003609.s003.docx]

1. prior receipt of influenza vaccination in the same season;
2. any serious active medical conditions including chronic disease of any body system,
3. chronic infections,
4. genetic disorders,
5. known or suspected disease of the immune system or chronic or active wheezing illness;
6. receipt of immunosuppressive agents including systemic corticosteroids during the month prior to study vaccination;
7. concomitant aspirin use;
8. history of Guillain-Barré Syndrome that occurred after receiving influenza vaccine in the past;
9. history of allergy to eggs or any of the vaccine ingredients; current or past participation (within two months of trial enrolment visit) in any clinical trial involving any investigational products;
10. history of a previous severe allergic reaction; or any condition determined by a study physician to be a significant potential health risk to the child.
