# Supplementary material for: Efficacy of live attenuated and inactivated influenza vaccines among children in rural India: A 2-year, randomized, triple-blind, placebo-controlled trial
Source: PLoS Med. 2021 Apr 29;18(4):e1003609. doi: 10.1371/journal.pmed.1003609 (PMC8118535; doi:10.1371/journal.pmed.1003609)
Supplement: S2 Text — (DOCX) [file pmed.1003609.s004.docx]

Viral RNA was extracted from samples using the MagMAX-96 Total RNA Isolation kit (Ambion, USA). A single step reverse transcription PCR was performed with the Superscript III Platinum One-Step qRT-PCR kit (Invitrogen, USA) for amplification of the entire HA gene, using primers described by the WHO for influenza A(H1N1)pdm09, and by the WHO Collaborating Center, Australia, for A/H3N2^[[1]](#endnote-1)^ (Deng YM *et al*, 2015) and B (personal communication). Amplicons for each fragment were visualized on a 2% agarose gel and purified using the Charge Switch PCR Clean-Up kit (Invitrogen,USA). DNA sequencing was carried out using the Big Dye Terminator v3.1 Cycle Sequencing kit (Applied Biosystems Inc., USA). Unincorporated labeled ddNTPs were purified using Dye Ex 2.0 Spin kit (Qiagen, USA). The sequencing was done on an ABI3730 DNA Analyzer (Applied Biosystems Inc., USA).

The MEGA ver. 6 software program was used for pair-wise sequence alignment and construction of neighbor-joining trees using Kimura’s two-parameter distance model, with 1000 bootstrap replicates. For phylogenetic analysis, HA gene sequences of influenza A(H1N1)pdm09 , H3N2 and B were compared with similar sequences from GenBank, including those of the corresponding WHO reference strains for the respective years

1. [↑](#endnote-ref-1)
