# Supplementary figures and images for: Efficacy of live attenuated and inactivated influenza vaccines among children in rural India: A 2-year, randomized, triple-blind, placebo-controlled trial
Source: PLoS Med. 2021 Apr 29;18(4):e1003609. doi: 10.1371/journal.pmed.1003609 (PMC8118535; doi:10.1371/journal.pmed.1003609)

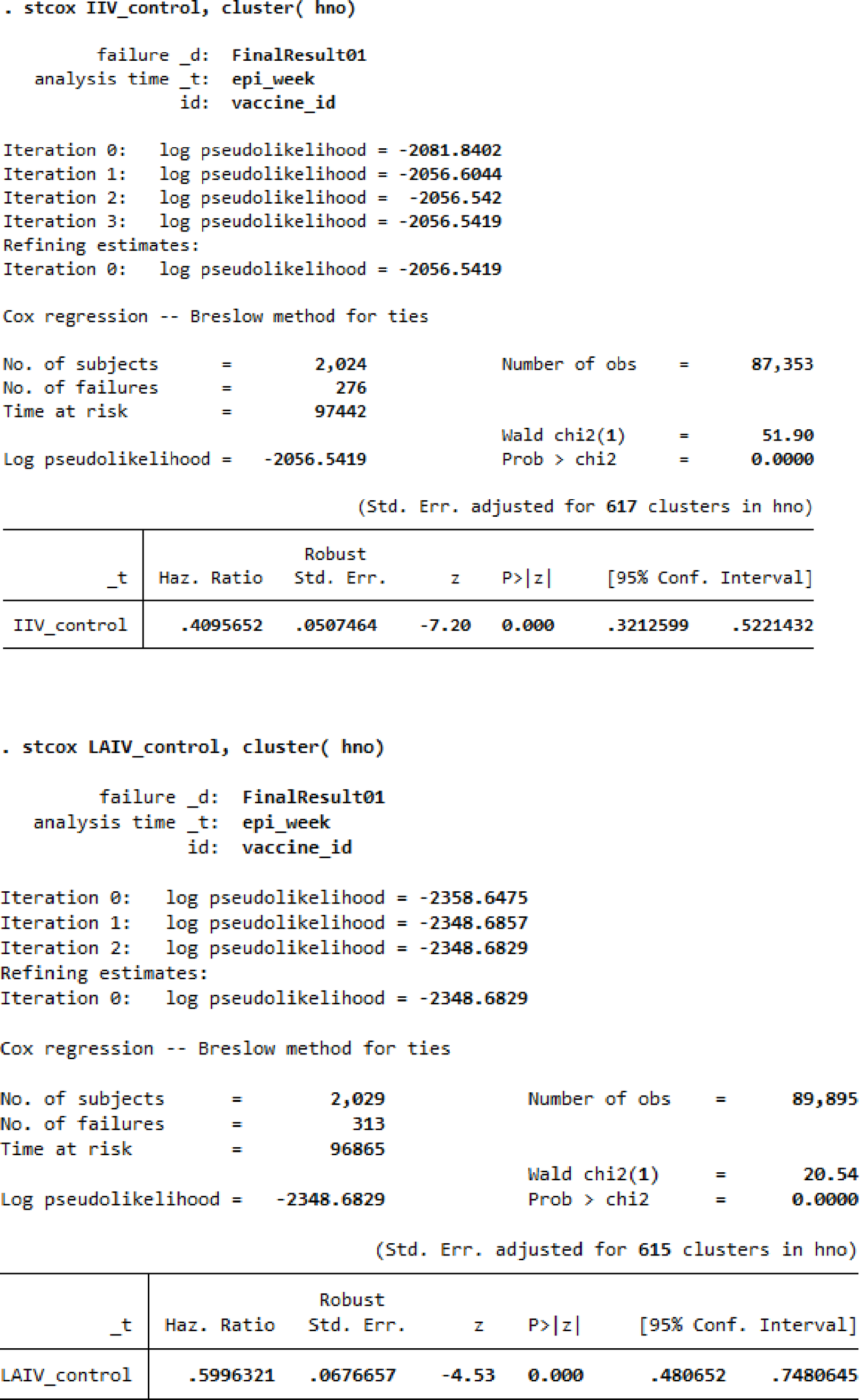

Supplement: S1 Fig — (TIF) [file pmed.1003609.s005.tif]

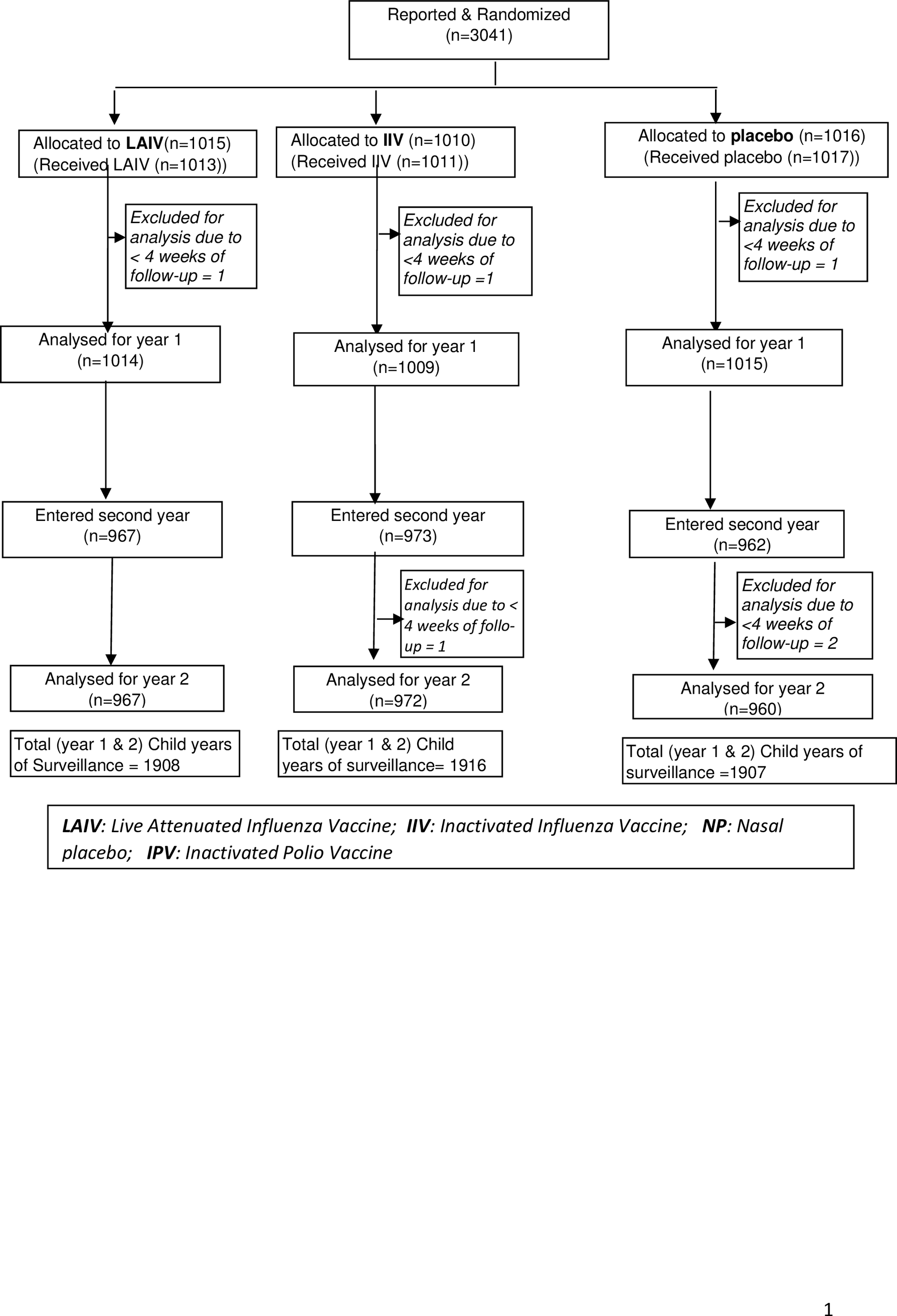

Supplement: S2 Fig — LAIV, live attenuated influenza vaccine; mITT, modified intention-to-treat. (TIF) [file pmed.1003609.s006.tif]

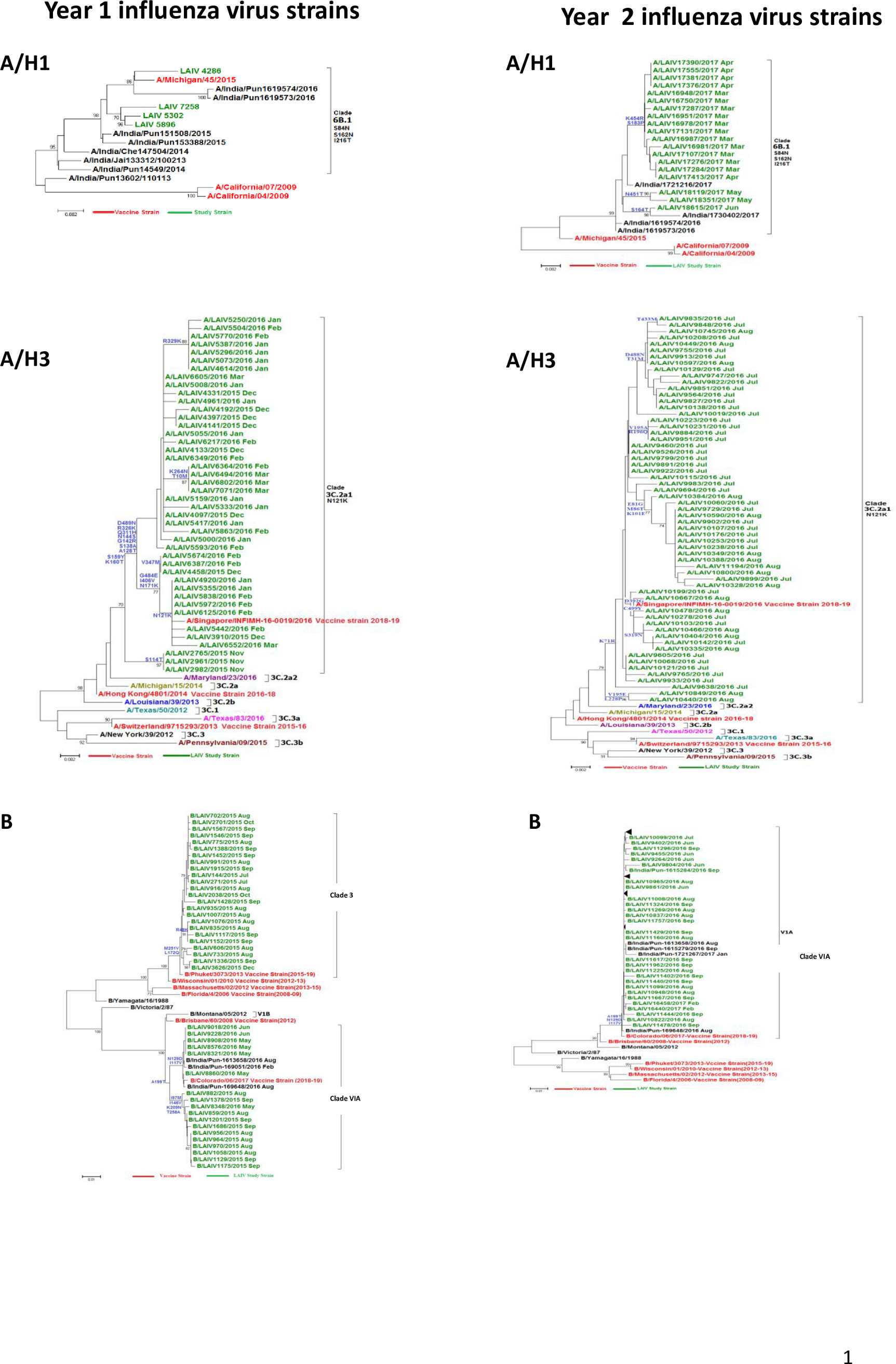

Supplement: S3 Fig — (TIF) [file pmed.1003609.s007.tif]
